# Nectin4-targeted molecular imaging in solid tumors: Current status and future perspectives

**DOI:** 10.1002/VIW.20250007

**Published:** 2025-04-16

**Authors:** Yongshun Liu, Wenpeng Huang, Jingwei Zhou, Rachel J. Saladin, Youlan Lei, Weibo Cai, Lei Kang

**Affiliations:** 1 Department of Nuclear Medicine, Peking University First Hospital, Beijing, China; 2 Department of Medical Imaging, Peking University First Hospital, Beijing, China; 3 Department of Plastic and Reconstructive Surgery, Shanghai Ninth People’s Hospital, Shanghai Jiao Tong University School of Medicine, Shanghai, China; 4 Departments of Radiology and Medical Physics, University of Wisconsin - Madison, Madison, Wisconsin, USA

**Keywords:** diagnostic, Nectin cell adhesion molecule 4, molecular imaging, theranostic, tumor

## Abstract

Nectin cell adhesion molecule 4 (Nectin4), a Ca^2+^-independent immunoglobulin-like cell adhesion molecule, plays a role in both physiological and pathological processes. Nectin4 is physiologically expressed at minimal levels in most normal adult tissues; however, it is significantly upregulated in various solid tumors. It can drive cell proliferation, metastasis, angiogenesis, adhesion, recurrence, and DNA mismatch repair in tumors, leading to poor prognosis. Notably, the United States Food and Drug Administration has approved enfutumab vedotin, a novel antibody–drug conjugate targeting Nectin4, for the therapy of urothelial carcinoma, highlighting the significance of Nectin4 in targeted therapy. However, accurate diagnosis and evaluation of patients is also important. Visualization of Nectin4 expression levels can be achieved with molecular imaging, including positron-emission tomography, single-photon emission computed tomography, and near-infrared fluorescence imaging. Incorporating Nectin4-targeted molecular imaging into clinical practice is vital for the diagnosis, differentiation, treatment decision, efficacy evaluation, and prognosis prediction of a broad spectrum of solid tumors. We reviewed research advances in Nectin4-targeted molecular imaging, focusing on theranostic applications in different tumor types, including breast, urothelial, gastric, pancreatic, ovarian, colorectal, and other cancers. We also evaluated the present situation by investigating research progress, diagnostic performance, and limitations. Finally, we discuss the challenges associated with clinical implementation and present our views on advancing this area to guide future research.

## 1 I INTRODUCTION

Nectin cell adhesion molecule 4 (Nectin4), also known as the poliovirus receptor-like 4 (PVRL4), is a type I transmembrane protein and belongs to the Nectin family.^1^ This transmembrane protein, composed of 510 amino acids, is divided into three distinct regions: a transmembrane domain, an extracellular domain, and a cytoplasmic tail.^2^ The extracellular domain is characterized by the presence of three loops resembling immunoglobulin structures, which include one V-type loop and two C2-type loops.^3^ The Nectin family is comprised of Nectins 1–4, which were first identified as viral entry receptors and named poliovirus receptor-related (PRR) proteins, but were later renamed for their role as Ca^2+^-independent immunoglobulin-like cell adhesion molecules.^4,5^ In the Nectin family, Nectins 1–3 are commonly enriched in normal adult tissues, but Nectin4 maintains a low abundance in most healthy adult tissues and circulates at negligible concentrations in serum.^6^ Nectin4 was initially only observed in embryonic and placental tissues, associated with ectodermal development.^7,8^ In healthy adults, it is normally expressed in a limited number of organ tissues, notably the esophagus, tonsils, and skin.^9^ It is noteworthy that both membrane-bound and soluble Nectin4 are abnormally enriched in various solid tumors, including lung,^10,11^ breast,^12–14^ urothelial,^15^ bladder,^16,17^ ovarian,^18^ gastric,^19^ pancreatic,^20^ head/neck,^21^ hepatocellular,^22^ and esophageal cancer.^23^ This upregulation is not merely incidental; Nectin4 is proven to be involved in tumor cell proliferation, metastasis,^24^ angiogenesis,^25^ adhesion,^26^ recurrence,^27^ DNA and mismatch repair,^28^ promoting disease progression. While the standalone diagnostic and prognostic significance of Nectin4 is limited, its distinctive expression pattern renders it an effective target for targeted therapy. Most importantly, the United States Food and Drug Administration (US FDA) has approved enfutumab vedotin (EV), an anti-Nectin4 antibody–drug conjugate (ADC), for the therapy of urothelial carcinoma (UC).^29^ Moreover, as a companion imaging modality for the assistance of EV therapy, Nectin4-targeted imaging holds substantial value. Nectin4 is thus recognized as a clinically validated and dependable antigen target for the development of effective cancer treatment strategies.^30^

Molecular imaging is the visualization and measurement of biological processes at biological systems’ molecular and cellular levels.^31^ The most commonly used molecular imaging techniques are nuclear medicine imaging techniques, such as positron emission tomography (PET) and single-photon emission computed tomography (SPECT) imaging. Optical imaging techniques, such as fluorescence (FL) and near-infrared (NIR) imaging, have various applications. Today, molecular imaging occupies a paramount position in theranostic applications across diverse solid tumor types, becoming a beacon of personalized medicine by enabling targeted diagnostic evaluation to inform the selection of specific treatments for patients.^32^ An emerging direction in this field is to explore Nectin4- targeted molecular imaging, especially immunoPET imaging, conducted in preclinical and clinical studies in some Nectin4-positive tumors. Molecular imaging relies on FL or radionuclides-labeled Nectin4-targeted monoclonal antibodies (mAbs), ADCs, nanobody–drug conjugates (NDCs), and bicyclic peptides as molecular probes to noninvasively monitor Nectin4 expression levels in tumors. Incorporating Nectin4-targeted molecular imaging into clinical practice is vital for optimizing EV therapy, particularly in identifying patients with high Nectin4 expression, monitoring treatment response, and guiding prognosis assessment in selected cases.

**FIGURE 1:**
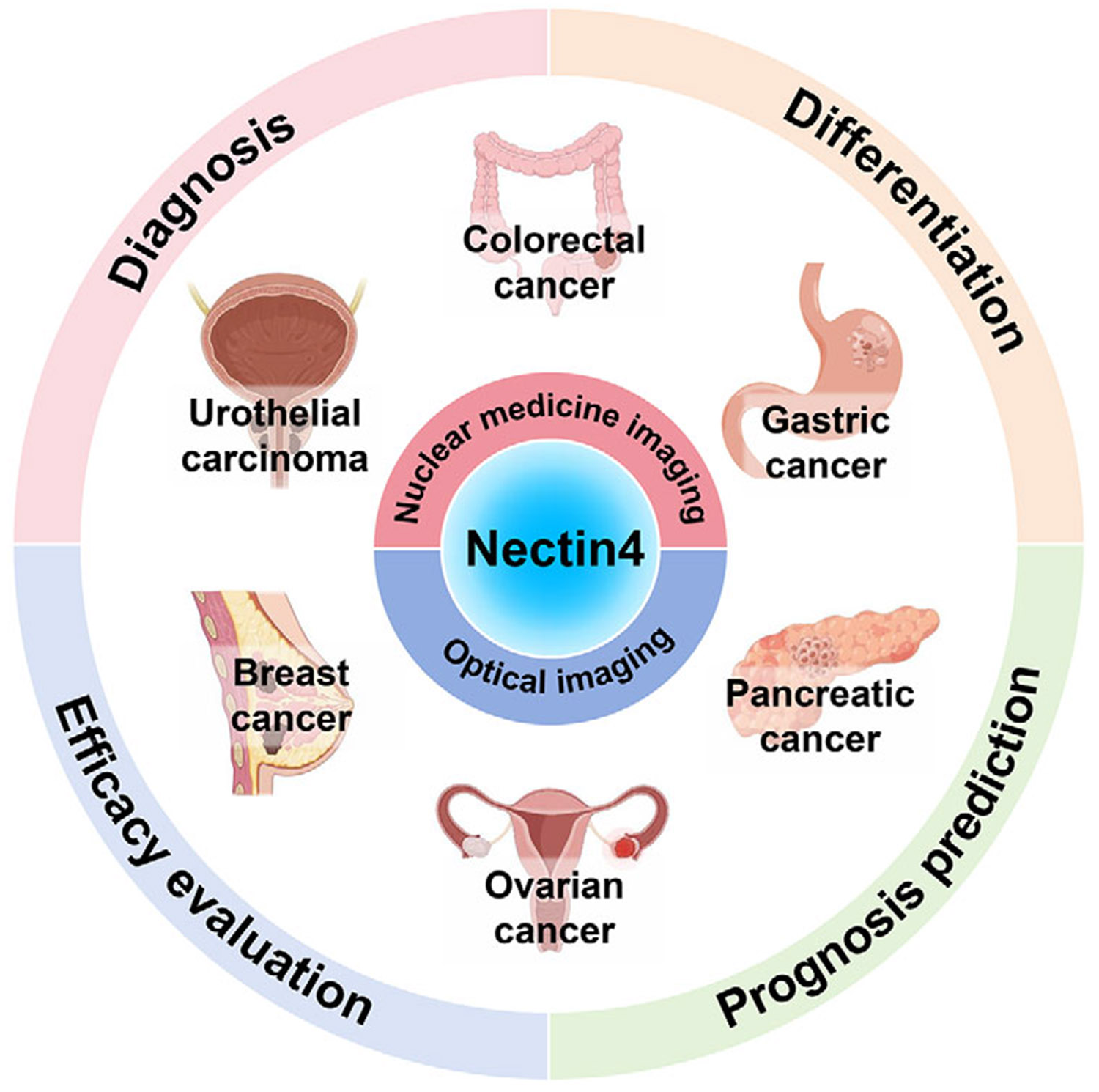
Nectin4-targeted molecular imaging in various tumors.

In this review, we first describe the mechanism of action of Nectin4 in various tumors and the targeted molecules used in the design of Nectin4-targeted probes. In addition, we discuss in depth the research progress, diagnostic performance, and limitations of Nectin4-targeted molecular imaging techniques including nuclear medicine and optical imaging in the diagnostic imaging of breast, urothelial, gastric, pancreatic, ovarian, colorectal, and other cancers (Figure 1). Finally, we discuss the challenges associated with clinical implementation and present our views on advancing this area to guide future research.

## 2 I MOLECULAR MECHANISM OF NECTIN4 IN CANCERS

**FIGURE 2:**
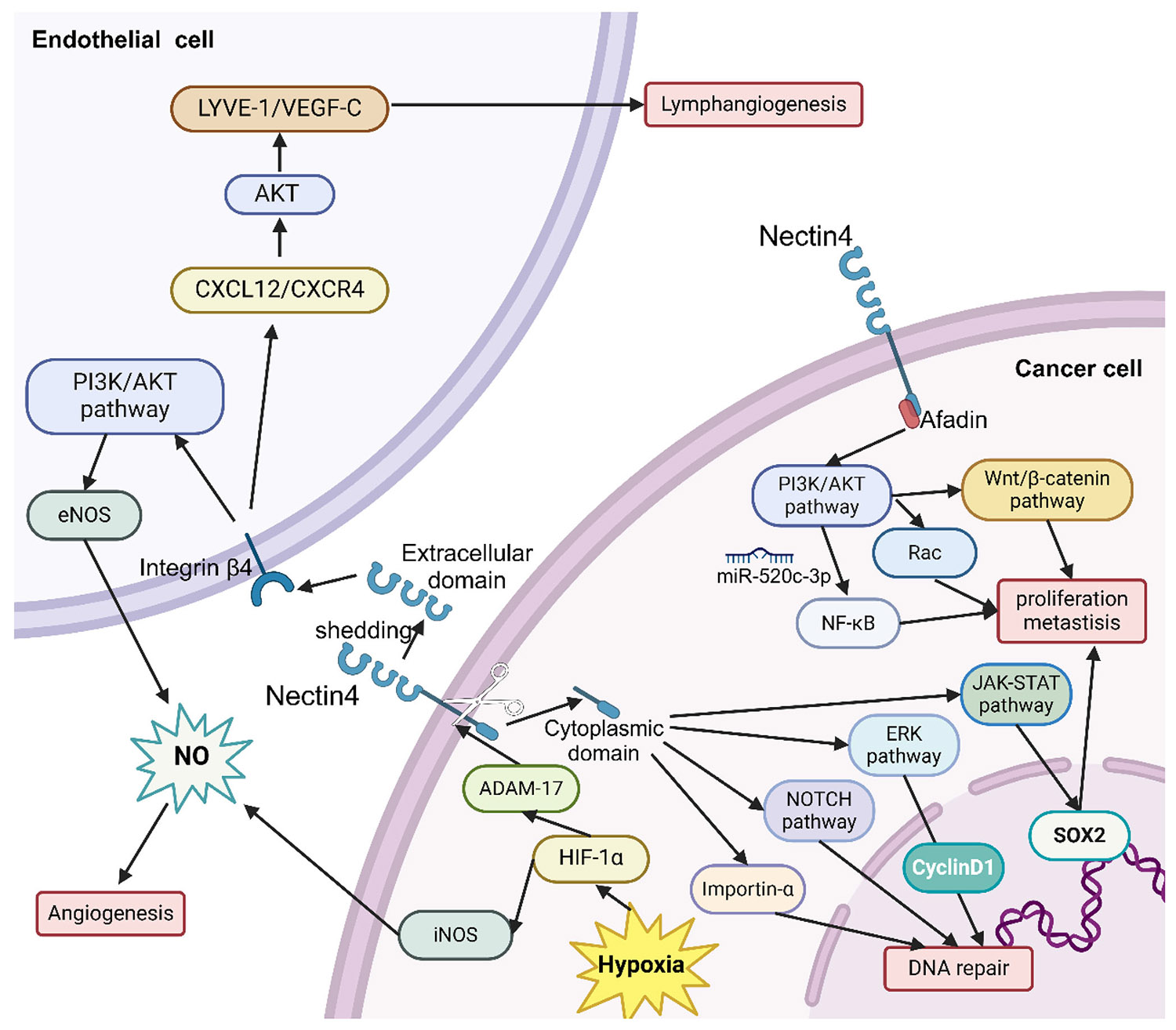
Nectin4 signaling network. Created in https://BioRender.com ERK, extracellular regulated protein kinases; PI3K/AKT, phosphatidylinositol 3-kinase/protein kinase B; NF-*κ*B, nuclear factor kappa-B; JAK-STAT, Janus kinase/signal transducer and activator of the transcription; ADAM-17, a disintegrin and metalloproteinase 17; HIF, hypoxia inducible factor; SOX2, sex-determining region Y-box 2; CXCR4, C-X-C motif chemokine receptor 4; CXCL12, stromal-derived-factor-1; LYVE, lymphatic vessel endothelial receptor.

Nectin4, a pivotal contributor in solid tumors, orchestrates a symphony of biological processes that include cell proliferation, metastasis,^24^ angiogenesis,^25^ adhesion,^26^ recurrence,^27^ DNA mismatch repair,^28^ and drug resistance.^33^ These key functions rely on Nectin4’s multifaceted involvement in various signaling pathways, emphasizing its potential importance as a cancer diagnosis and treatment research target. We have summarized multiple signaling pathways in which Nectin4 participates in tumor progression (Figure 2).

The phosphatidylinositol 3-kinase (PI3K)/protein kinase B (PKB/AKT) signaling pathway is a key signaling pathway for Nectin4 to participate in tumor development. Nectin4 can interact with certain proteins to activate this pathway. Nectin4 can form a complex with Afadin, an F-actin binding protein, to establish intercellular connections,^34^ activate the PI3K/AKT pathway,^7,35^ enhance tumor cell survival, and inhibit apoptosis.^33^ Specifically, the cis interaction between Nectin4 and Her2, a receptor tyrosine kinase (RTK), can also activate this pathway, promoting DNA synthesis in breast cancer.^36^ In addition, the extracellular domain of Nectin4 can be cut into soluble Nectin4 by a disintegrin and metalloproteinase 17 (ADAM-17) under hypoxia conditions.^9^ Combined with endothelial Integrin-*β*4, it can activate the PI3K/AKT pathway, mediate iNOS or eNOS cascade reaction in breast cancer and oral cancer, and promote tumor angiogenesis.^37,38^

The PI3K/AKT pathway can also promote tumor proliferation and metastasis by affecting other molecular pathways. The Wnt/*β*-catenin signaling pathway is crucial for regulating epithelial–mesenchymal transition and can regulate self-renewal of cancer stem cells.^39^ It was found that soluble Nectin4 can induce the expression of the Wnt/*β*-catenin pathway through the PI3K/AKT pathway and promote cell proliferation and metastasis in breast cancer.^40^ The precise mechanism could involve the facilitation of INPP4B-mediated lysosomal degradation by PI3K*α* signaling, which in turn activates the Wnt/*β*-catenin pathway.^41^ The nuclear factor kappa-B (NF-*κ*B) signaling pathway is essential in cellular signaling, playing a crucial role in regulating various processes such as immune response, inflammatory response, and cell apoptosis.^42^ Nectin4 also downregulates miR-520c-3p levels that directly target AKT-1, activates the NF-*κ*B pathway through the PI3K/AKT pathway, and promotes the progression and metastasis of osteosarcoma.^43^ The activation mechanism of this pathway may be that AKT regulates NF-*κ*B signaling transduction by phosphorylating IKK*α* and Tpl2.^44^ Nectin4 specifically stimulates the activation of Ras-related C3 botulinum toxin substrate 1 (Rac1) via the PI3K/AKT signaling pathway, thereby facilitating cell proliferation and metastasis in gallbladder cancer.^24^ Overall, Nectin4 can interact with various upstream and downstream targets through the PI3K/AKT signaling pathway, playing an important role in tumor development.

In addition to the PI3K/AKT pathway, Nectin4 is also involved in the transduction of other signaling pathways. The Notch signaling pathway mediates the activation effect of two cells after close contact by using membrane proteins as ligands and receptors.^45^ Nectin4 induces DNA mismatch repair (BER) in 5-FU-resistant cervical cancer by regulating the ADAM-17-mediated Notch signaling pathway.^46^ In addition, Nectin4 can also participate in tumor formation through the Janus kinase/signal transducer and activator of the transcription (JAK–STAT) signaling pathway. The JAK–STAT signaling pathway is an evolutionarily conserved signaling pathway activated by cytokine stimulation, which transmits extracellular signals through the cell membrane to the nucleus, causing changes in DNA transcription.^47^ The development of mammary follicles relies on the extracellular domain of Nectin4 and the cis interaction of prolactin receptors, which depends on the JAK2–STAT5a signaling pathway.^48^ The JAK2–STAT5a pathway is critical to regulating the growth of certain malignancies, including breast cancer and prostate cancer.^49,50^ The JAK–STAT3 signaling pathway can also be activated by p95–ErbB2, which is induced by Nectin4.^36^ This pathway works in conjunction with p95–ErbB2 to coregulate the expression of the sex-determining region Y-box 2 (SOX2) gene. The regulation of SOX2 is significant as it influences the proliferation, survival, and differentiation of cancer cells, ultimately promoting the proliferation of breast cancer cells.^51^ More over, Nectin4 can stimulate the expression of C-X-C motif chemokine receptor 4 (CXCR4) and stromal-derived-factor-1 (SDF-1, also known as CXCL12) in breast cancer under hypoxia conditions, regulate the CXCR4/CXCL12– lymphatic vessel endothelial receptor-1 (LYVE-1) axis, and assume a pivotal position in facilitating tumor-driven lymphangiogenesis and lymphatic migration.^52^ In particular, Nectin4 may regulate tumor progression through the extracellular regulated protein kinase (ERK) signaling pathway. Tanaka et al.^53^ knocked out Nectin4 in cutaneous squamous cell carcinoma cells and found a decrease in ERK phosphorylation. There is also a notable reduction in cyclin D1 expression and a decrease in cell proliferation. The same results can also be observed when using ERK inhibitors. These prove that Nectin4 can promote the proliferation and migration of squamous cell carcinoma cells through the modulation of the ERK signaling pathway.

It is worth noting that Nectin4 can also directly act on certain cells and participate in tumor progression. It was found that Nectin4 can interact with the inhibitory checkpoint receptor TIGIT to inhibit natural killer (NK) cell activity and promote cancer progression.^54^ It has also been reported that Nectin4 enhances vasodilation and triggers angiogenesis by converting endothelial stalk cells into tip cells.^38^ In addition, Nectin4-endo-domain cleaved by ADAM-17 engages in an interaction with IMPORTIN- *α*2, facilitating its translocation into the nucleus. This process not only initiates DNA repair mechanisms but also enhances the proliferation of cancer cells.^55^ The results underscore the complex role of Nectin4 in multiple molecular pathways that facilitate tumor progression, thereby advancing the investigation of its viability as a target for cancer diagnosis and therapeutic interventions.

## 3 I NECTIN4-TARGETED MOLECULES FOR MOLECULAR PROBE DESIGN

Molecular probes are the cornerstone of molecular imaging, and they are important tools for visualizing and quantifying biological processes at the molecular levels.^56^ The Nectin4-targeted molecular probes, which use radioactive isotopes or fluorescent labeling of Nectin4-targeted molecules, have immense value as a tracer or targeted drug in cancer imaging diagnosis, prognosis prediction, and therapy research. The currently developed Nectin4-targeted molecules include mAbs, ADCs, NDCs, and bicyclic peptides. Molecular probes based on these targeted molecules have been used in preclinical experiments and clinical studies, and they will have greater applications and value in the future. We have summarized the Nectin4-targeted molecules currently used for designing molecular probes in research and briefly introduced their concepts and applications.

### 3.1 I Monoclonal antibodies

mAbs are immunoglobulins produced by only one type of immune cell and target only a specific antigenic epitope. Their precision and effectiveness are important in targeted therapy for malignant tumors and immune diseases.^57^ Currently, few studies use mAbs alone for Nectin4-targeted therapy, as most studies combine mAbs with drugs to form ADCs. Individual mAbs are predominantly utilized in molecular imaging due to their high specificity and affinity for target antigens. Nevertheless, their substantial molecular weight prolongs circulation time within the bloodstream, potentially limiting their efficacy in scenarios requiring rapid localization or clearance.^58^

### 3.2 I Antibody–drug conjugates

ADCs show exciting potential in targeted drug delivery by linking mAbs targeting Nectin4 with cytotoxic drugs, delivering the drugs near tumors highly expressing Nectin4. This targeted delivery method makes the drug much more specific than normal drugs.^59^ Multiple ADCs exist in the preclinical and clinical stages of Nectin4 targeted therapy research.^30^ Most importantly, the US FDA has approved EV, a novel ADC targeting Nectin4, for treating UC.^29^ EV consists of a humanized IgG1 mAb targeting Nectin4 and a microtubule inhibitor monomethyl auristatin E (MMAE), and these two components are connected by a protease cleavable linker, maleimide caproyl-valine-citrulline (MC Val Kit).^29^ In particular, EV can not only be used for targeted therapy but can also be labeled with ^124/125^I as a tracer for nuclear medicine imaging.^60^ All of these indicate that ADC targeting Nectin4 is an important therapeutic tool for solid tumors and has great potential for the diagnostic application of tumors.

### 3.3 I Nanobody–drug conjugates

NDC delivers drugs to tumors specifically labeled with antibodies by linking nanobodies with cytotoxic drugs. Due to the smaller volume of nanobodies compared with mAbs, NDCs have higher tissue penetration and stronger cell-killing ability. Meanwhile, NDC has a faster clearance rate and stronger stability.^61^ At present, the preliminary study has explored the feasibility of targeted therapy for gastric cancer with Nectin4 NDC. It has been found that Nectin4 NDC has ideal in vitro cell binding activity and cytotoxic effect on cells with high expression of Nectin4. In NCI-N87 human gastric cancer xenograft mice, Nectin4 NDC shows rapid tissue penetration and high tumor uptake, demonstrating its potential for targeted therapy of gastric cancer. In addition, it is speculated that the binding of Nectin4 NDC to NIR fluorescence (NIRF) may serve as a specialized tracer for cancer imaging. This indicates that Nectin4 NDC has great potential for tumor diagnosis and treatment development, and future research in this area may be more in-depth.

### 3.4 I Bicyclic peptides

Bicyclic peptides are small, constrained peptides with two cyclic peptide molecules.^62^ Polypeptide drugs have the advantages of high safety and selectivity, but their oral bioavailability and stability are poor.^63^ Cyclic peptide structure can improve these shortcomings, such as enhancing metabolic stability and enhancing target affinity. Bicyclic peptides have rapid tissue penetration similar to small molecule drugs, and targeting similar to antibody drugs.^64^ The Nectin4-targeted bicyclic peptide can be conjugated to the cytotoxin MMAE via a cleavable linker to give the peptide–drug conjugate, BT8009.^65^ It has shown significant anti-tumor activity and good tolerability in preclinical studies and is currently undergoing phase III clinical trials.^66^ In addition, one Nectin4-targeted bicycle can be conjugated with two CD137-targeted bicycles through a series of polyethylene glycol (PEG)-based linkers to form BT7480. BT7480, as a bispecific molecule, can selectively target Nectin4-positive tumor cells while simultaneously activating CD137 on adjacent immune cells. This dual action serves to modify the tumor immune microenvironment, leading to the destruction of tumor cells and the prevention of tumor recurrence.^67,68^ Previous studies have used ^68^Ga-labeled Nectin4-targeted bicyclic peptides as tracers for PET/CT imaging of different solid tumors. They found that ^68^Ga–N188, ^68^Ga–NOTA–PEG_12_–BP, and ^68^Ga– DN68 bicyclic peptide molecular probes, can effectively monitor the expression level of Nectin4 in tumors. These demonstrate the vital role of Nectin4-targeted bicyclic peptide in tumor diagnostic imaging and targeted therapy.

The Nectin4-targeted molecules described above can be labeled with radioactive isotopes for nuclear medicine imaging. When choosing the radioactive isotope, factors such as the molecular weight of the molecular probe, its clearance efficiency, and its ability to bind to the target must be considered.^69^ Among them, the mAb probe has the advantages of high stability and specificity.^70^ However, the large molecular weight of mAb results in a longer clearance period in vivo and a longer waiting time to obtain the ideal image.^71^ Therefore, the mAb probes generally need to be matched with radioisotopes with long half-lives, such as ^89^Zr (half-life 3.3 days) and ^124^l (half-life 4.2 days).^72,73^ NDC and bicyclic peptides have smaller molecular weights, higher clearance efficiency in vivo, and short duration, so they are generally matched with shorter half-life radioactive isotopes compared with mAb probes, such as ^68^Ga (half-life 67.6 min), which require less observation time in clinical application.^74^ Future research directions may focus on the development of smaller molecular size probes, such as nanobodies and bicyclic peptides, which retain high affinity and specific binding ability while overcoming limitations, such as prolonged circulation half-lives and inconsistent clinical results.^75^

## 4 I NECTIN4-TARGETED MOLECULAR IMAGING

Due to its limited expression in healthy adult tissues and overexpression in many solid tumors, Nectin4 can be a target for diagnosing and detecting disease progression.^28^ Nectin4-targeted molecular imaging provides an accurate way to diagnose and treat a variety of tumors, which relies on specific targeted diagnostic tests to help screen patients suitable for targeted therapy, primarily including optical and nuclear medicine imaging.

Optical imaging has been widely used in biological detection as a simple, intuitive, and in situ visual observation technique.^76^ In particular, NIRF imaging is the most commonly used technology for Nextin4-targeted optical imaging. NIRF imaging is based on the infrared spectrum, which captures the NIR light generated by FL probe excitation through the imaging system and forms real-time images through advanced data processing technology.^77^ Since the NIR spectral region is nonvisible light, it has a low absorption and scattering rate in biological tissues, so it has deeper tissue penetration and significantly increases the imaging depth.^78^ Moreover, the autofluorescence of biological tissue is less in this band, so the imaging has a higher signal-to-noise ratio and sensitivity.^79^ NIR combined with intraoperative staining allows real-time FL images to provide accurate guidance during surgery.^80^

Nuclear medicine imaging is a technique that uses radionuclide-labeled molecular probes for imaging, mainly including PET and SPECT.^81^ It has the advantages of high specificity, sensitivity, repeatability, safety, and noninvasiveness.^82^ Unlike conventional nuclear medicine imaging, which uses nontargeted ^18^F-FDG as a tracer, Nectin4-targeted nuclear medicine imaging uses targeted molecules (such as antibodies and peptides) labeled by radionuclides as tracers. This can improve the specificity of molecular imaging and marks a paradigm shift in molecular imaging patterns.^83^ Currently, Nectin4-targeted molecules include mAbs, ADCs, and NDCs, bicyclic peptides. They are ideal for metabolic and functional imaging applications with extremely high sensitivity and resolution.

Therefore, we adopted the following search strategy and finally selected nine articles on the application of Nectin4-targeted molecular imaging in the breast, urothelial, gastric, pancreatic, and other cancers (Table 1): A comprehensive examination was carried out using the PubMed/MEDLINE and Cochrane Library databases, with the search extending until November 20, 2024. The search keywords were as follows: (A) “Breast” OR “Mammary” OR “Urothelial” OR “Urothelium” OR “Bladder” OR “Gastric” OR “Gastrointestinal” OR “Digestive” OR “Pancreatic” OR “Pancreas” AND (B) “Tumor” OR “Tumour” OR “Carcinoma” OR “Neoplasm” OR “Cancer” AND (C) “Nectin4” OR “Nectin” OR “Nectin cell adhesion molecule 4” OR “PVRL4” OR “Poliovirus receptor-like 4” OR “PRR4” OR “Poliovirus receptor-related 4” AND (D) “Imaging” OR “PET” OR “PET/CT” OR “SPECT” OR “Fluorescence.” The database search was performed without a defined temporal constraint, focusing exclusively on studies published in English. Authors of articles that were not available online were not approached for additional manual retrieval. In their commitment to rigorous selection criteria, researchers Liu and Huang diligently reviewed a vast array of references, and they ultimately selected a refined set of nine high-quality articles for their investigation. These articles were classified based on tumor types, with a summary of the applications, diagnostic potential, and limitations of molecular imaging in oncology. Furthermore, the study addressed the challenges related to clinical implementation and offered perspectives on future advancements in this domain.

### 4.1 I Breast cancer

Breast cancer is one of the three most common cancers worldwide, especially among women, and is the second leading cause of cancer-related deaths on a global scale.^84^ Triple-negative breast cancer (TNBC), is known for its aggressiveness, high likelihood of recurrence, and poor prognosis.^85^ It is characterized by a lack of human epidermal growth factor receptor 2, estrogen receptor, and progesterone receptor expression.^86^ Nectin4 is upregulated in the majority of breast cancer cases and is associated with tumor size, grade, lymph node infiltration, angiogenesis, and lymphatic metastasis, leading to poor patient survival rates.^87,88^ Overexpression of Nectin4 was also found in TNBC, which was associated with poor prognosis such as low patient survival as well.^13,14^ Currently, immunoPET and SPECT are used to detect Nectin4 expression levels in breast cancer, enabling noninvasive imaging and accurate diagnosis and classification.

In 2016, Dean et al.^89^ used radioactive isotopes ^89^Zr to label AGS-22M6, a Nectin4-targeted mAb, as an immunoPET probe first used in molecular imaging. The isotope ^89^Zr, with a long half-life of 3.3 days, is suitable for labeling large molecules with low clearance efficiency in vivo, such as mAbs.^90^ Generally, it can be used for long-term imaging, and due to the outstanding positron emission, it has an excellent imaging effect for PET.^91^ To study the specificity of ^89^Zr–AGS-22M6 to Nectin4, they compared the uptake of ^89^Zr–AGS-22M6 PET in Nectin4-positive MDA–MB-231–Nectin4 and Nectin4-negative MDA–MB-231–Neo mice bearing tumors. It was found that ^89^Zr–AGS-22M6 has a higher uptake in MD– MB-231–Nectin4 tumors in vivo and ex vivo, suggesting that this probe could be used to distinguish breast cancers based on Nectin4 expression. Then, they still used MDA– MB-231–Nectin4/Neo isogenic pairs to simulate metastatic lesions in mouse liver and tibia and evaluated ^89^Zr– AGS-22M6’s ability to detect metastatic lesions. In liver tumor research, Nectin4-positive tumors were identified earlier and displayed a gradual rise in PET signal intensity, reaching a peak of approximately 98.8% ID/g by day 7. In contrast, Nectin4-negative tumors peaked earlier (around 27.4% ID/g at day 2) before decreasing. The ^89^Zr–AGS-22M6 imaging agent successfully differentiated Nectin4 expression despite interference from liver tissue signals. For bone tumors, Nectin4-positive lesions showed markedly higher activity (66.56% ID/g) compared with negative ones (11.94% ID/g), demonstrating 5.6-fold specificity for bone metastases by day 3. Monkey studies with ^18^ F-AGS-22M6 revealed minimal nontarget background activity, improving lesion visibility. This evidence supports Nectin4-targeted immunoPET as a valuable method for detecting tumors, categorizing patients, and tracking disease progression.

**FIGURE 3:**
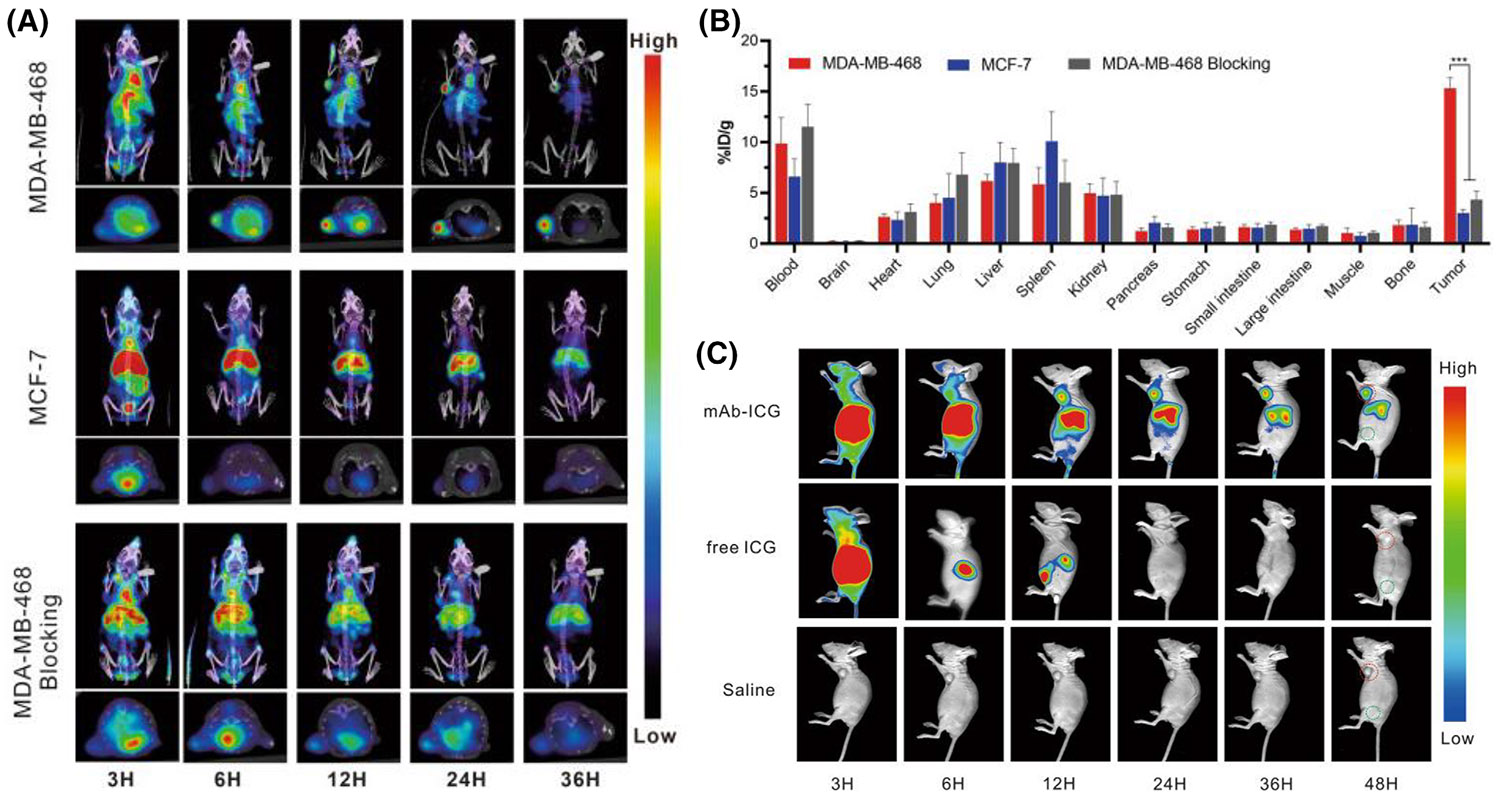
SPECT and NIRF imaging enable visualize Nectin4-positive breast cancers. (A) ^99m^Tc–HYNIC–mAb_Nectin4_ SPECT/CT imaging in cancer cells. (B) Biodistribution of ^99m^Tc–HYNIC–mAb_Nectin4_ in different organs and tumors at 36 h p.i. (*n* = 3). (C) NIRF imaging for MDA–MB-468 tumor-bearing mice. Red circles indicate the tumor sites and green circles indicate the normal muscles (*n* = 3). Reproduced under the terms of the CC-BY 4.0 license.^92^ Copyright 2022, Springer. SPECT, single-photon emission computed tomography; NIRF, near-infrared fluorescence; mAb, monoclonal antibody.

After the development of the first Nectin4-targeted probe for imaging, Shao et al.^92^ used another radionuclide, ^99m^Tc, to label anti-Nectin4 mAb for SPECT imaging of TNBC. The isotope ^99m^Tc, with a short half-life of 6.02 h, is produced by the ^99^Mo-^99m^Tc generator.^93^ Due to its ideal developer properties including high affinity, low toxicity, and single gamma-ray emission, ^99m^Tc has been widely used in SPECT imaging.^94,95^ First, they compared the uptake of the molecular probe in three groups of cells (MDA–MB-468, MCF-7, MDA–MB-468 blocking), and found that ^99m^Tc–HYNIC–mAb_Nectin4_ had a significant ability to target Nectin4-positive cells in vitro. Then, they performed microSPECT/CT imaging on the three groups of tumor-bearing mice and found that the uptake of ^99m^Tc–HYNIC–mAb_Nectin4_ in Nectin4-positive tumors was higher than the control group and the blocking group (Figure 3A,B), demonstrating the probe’s ability to target Nectin4 in vivo. They further designed a mAb_Nectin4_-based fluorescent probe (mAb_Nectin4_–ICG) as a photothermal agent for TNBC xenograft photothermal therapy (PTT). ICG is a US FDA-approved NIRF dye with an excitation range of 700–900 nm, low toxicity, and rapid metabolism.^96^ To verify the in vitro targeting of mAb_Nectin4_–ICG, they incubated MDA–MB-468 cells with three groups, mAb_Nectin4_–ICG, free ICG, or saline, respectively, and then performed NIRF imaging. The cells with mAb_Nectin4_–ICG had significant FL uptake, while the cells in the other groups had only a weak FL signal (Figure 3C). This suggests that mAb_Nectin4_–ICG can target cells with high Nectin4 expression. In addition, they used mAb_Nectin4_–ICG FL signals to determine the optimal time for PTT treatment in the same three groups of mice carrying MDA–MB-468 TNBC tumors. They captured tumor FL signals only in the mAb_Nectin4_–ICG group, indicating that mAb_Nectin4_–ICG has good tumor-specific targeting and long-term retention. In summary, ^99m^Tc–HYNIC– mAb_Nectin4_ SPECT/CT imaging helps characterize Nectin4 expression in TNBC and facilitates accurate diagnosis and classification. After stratification by Nectin4 targeted imaging, mAb_Nectin4_–ICG-mediated PTT is an emerging drug for treating Nectin4-positive tumors. These diagnostic and therapeutic pairs have immense potential to improve the management of TNBC patients.

**FIGURE 4:**
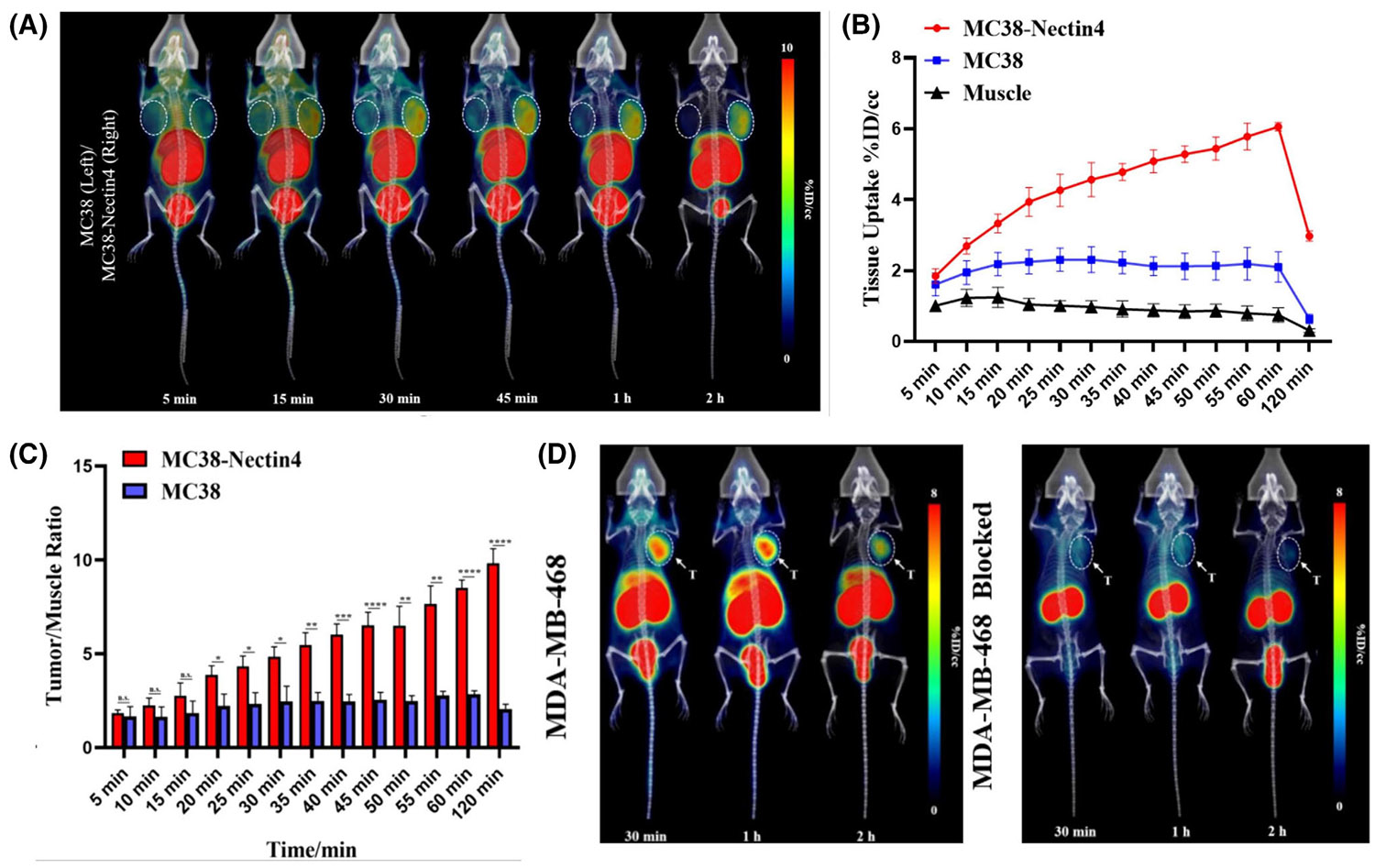
^68^Ga–DN68 PET/CT images and imaging analysis in breast cancer. (A) ^68^Ga–DN68 PET/CT images in MC38–Nectin4 and MC38 tumor-bearing mice. (B) The uptake of ^68^Ga–DN68 in tumor-bearing mice. (C) The T/M ratios of two tumor models. (D) ^68^Ga–DN68 PET/CT images in MDA–MB-468 tumor-bearing mice with or without blocking. Reproduced with permission.^97^ Copyright 2024, Elsevier. PET, positron-emission tomography.

Recently, Ge et al.^97^ developed a novel ^68^Ga-labeled bicyclic peptide molecular probe targeting Nectin4, designated ^68^Ga–DN68. The isotope ^68^Ga, with a short half-life of 67.6 min, is suitable for labeling small molecule drugs and polypeptide tracers.^74^ The clinical acquisition is produced through Ge-68/Ga-68 generators, making it ideal for popularization.^98^ In vitro experiments, they compared the cell uptake in MC38–Nectin4, MC38, and MC38–Nectin4-blocking groups, and found that the ^68^Ga–DN68 uptake in MC38–Nectin4 cells was significantly higher than in other groups. This demonstrated the high affinity and specificity of ^68^Ga–DN68 for Nectin4. Then, they performed dynamic PET scans of mice with MC38–Nectin4/MC38 tumors (Figure 4A) and found that ^68^Ga–DN68 rapidly accumulated in MC38–Nectin4 tumors. Tumor uptake of MC38–Nectin4 tumors peaked at 60 min (6.06 ± 0.12% ID/cc), which is a much higher value than that of MC38 tumors (2.10 ± 0.43% ID/cc) (Figure 4B). In addition, only the tumor/muscle target ratio in the MC38–Nectin4 group increased gradually over time, reaching a maximum of 9.83 ± 0.77 at 2 h (Figure 4C). Similarly, they performed PET/CT in MDA–MB-468 xenograft tumors and MDA–MB-468 blocked tumors (Figure 4D). They found that MDA–MB-468 tumors also had a higher ^68^Ga–DN68 uptake than MC38 tumors and excessive nonradioactive compound DN68 reduced tumor uptake of ^68^Ga–DN68. In addition, biodistribution analysis showed that ^68^Ga– DN68 was metabolized mainly by the kidney, followed by the liver. In particular, ^68^Ga–DN68 demonstrated a high target-to-nontarget ratio, exceeding 20 at 1 h. The above data all indicate that ^68^Ga–DN68 is an excellent molecular probe and shows great potential for Nectin4 noninvasive detection in TNBC.

Overall, relevant studies have demonstrated the potential of Nectin4-targeted mAbs and bicyclic peptides in the theranostic of breast cancer. Future research directions may be to develop smaller probes, such as bicyclic peptides, which have high clearance efficiency in the body and are conducive to clinical application.

### 4.2 I Urothelial carcinoma

UC is the 6th most common cancer and the second most common genitourinary tract cancer.^99^ Most UCs originate in the bladder and account for more than 90% of bladder cancers.^100^ It was found that the expression of Nectin4 is upregulated in most UCs and associated with poor prognosis.^101^ Recently, a Nectin4-targeted ADC, EV, has been approved by the US FDA for the treatment of metastatic UC, marking a significant achievement of Nectin4-targeted therapy.^29^ In addition, Nectin4-targeted PET/CT in UC has shown potential for disease diagnosis and monitoring of Nectin4 expression in preclinical and clinical studies.

Duan et al.^102^ developed ^68^Ga–N188, a PET tracer targeting Nectin4, and evaluated its performance in both preclinical and clinical settings. In preclinical studies using mice, the tracer exhibited significantly higher uptake in Nectin4-positive tumors compared with blocked or low- expressing groups, achieving a tumor-to-muscle ratio of approximately 4. Then they performed ^68^Ga–N188 PET/CT in 2 healthy volunteers and 14 patients with advanced UC and found that uptake of ^68^Ga–N188 is consistent with IHC and literature reports of Nectin4 distribution in vivo.^103^ When compared with ^18^F-FDG PET/CT imaging, ^68^Ga–N188 had significantly lower background accumulation in the brain, heart, and liver than ^18^F-FDG, and could be used as a complementary imaging method for ^18^F-FDG. They also found that higher Nectin4 levels in tumors correlated with increased ^68^Ga–N188 uptake on PET scans. In a follow-up trial with 16 UC patients, scans using ^68^Ga–N188 also linked stronger signals to high Nectin4 expression.^104^ These results suggest the tracer could help identify patients with Nectin4-rich tumors for targeted therapy and predict treatment outcomes. Researchers also analyzed imaging timing by tracking tumor activity over time and comparing ^68^Ga–N188 to standard ^18^F-FDG scans. The ^68^Ga–N188 method showed reliable accuracy in detecting Nectin4-positive lesions. While effective for diagnosis, the probe’s moderate tumor absorption highlights the need for structural improvements to boost its targeting efficiency.

**FIGURE 5:**
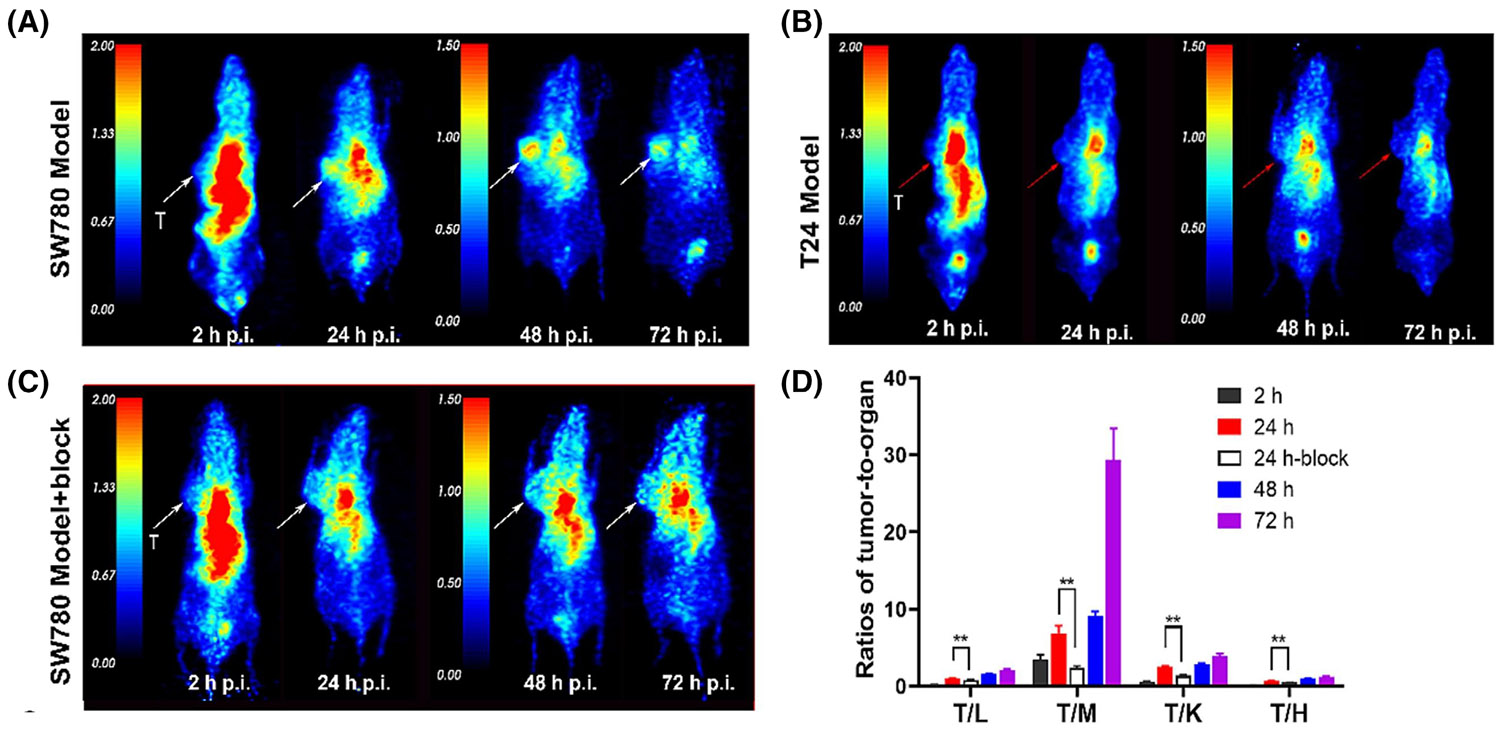
^124^I-EV PET/CT imaging enables visualize UC. PET images of ^124^I-EV in the SW780 xenograft model without blocking (A) and with blocking (B) and in the T24 xenograft model (C). The SW780 and T24 tumors are denoted by white and red arrows, respectively. (D) Ratios of tumor-to-organ according to ROIs. Reproduced with permission.^60^ Copyright 2024, Elsevier. PET, positron-emission tomography; UC, urothelial carcinoma; ROI, region of interest.

Recently, Ren et al.^60^ labeled EV with radionuclide ^124^I as a novel ADC PET probe with the high specificity and binding affinity of Nectin4. The isotope ^124^I, with a long half-life of 4.2 days, has great potential in ADC labeling as its simple radiolabeling process.^105^ They performed PET/CT imaging in the Nectin4-positive SW780 xenograft model without blocking or with blocking, and Nectin4-negative T24 xenograft tumor models (Figure 5A–C). The accumulation of ^124^I-EV was significantly higher in SW780 tumors (SUVmax of 1.50 ± 0.01 at 24 h p.i.) than in T24 tumors (SUVmax of 0.52 ± 0.02 at 24 h p.i., *p* < .001). Additionally, the absorption of ^124^I-EV in the SW780 tumor was markedly inhibited when it was coadministered with unlabeled EV, resulting in a reduction of the SUVmax to 0.94 ± 0.05 at 24 h postinjection (*p* = .005). Further-more, favorable target-to-nontarget ratios were achieved, attributed to the selective uptake in tumors and minimal accumulation in nontarget organs (Figure 5D). These results indicate that ^124^I-EV can identify Nectin4 positive and negative tumors. Compared with the previous bicyclic peptide probe ^68^Ga–N188, ^124^I-EV showed ideal Nectin4 tumor targeting. The *K*_d_ value of ^124^I-EV (13.8 nM) was lower than ^68^Ga–N188 (23.7 nM), and the highest SUVmax value of ^124^I-EV (1.50 ± 0.01 at 24 h p.i.) was higher than ^68^Ga–N188 (<1 at 1 h p.i.) in SW780 tumors.^102^ All these suggest that the ^124^I-EV PET/CT can be used to diagnose UC and visualize Nectin4 expression, establishing a foundational classification for targeted therapies.

**FIGURE 6:**
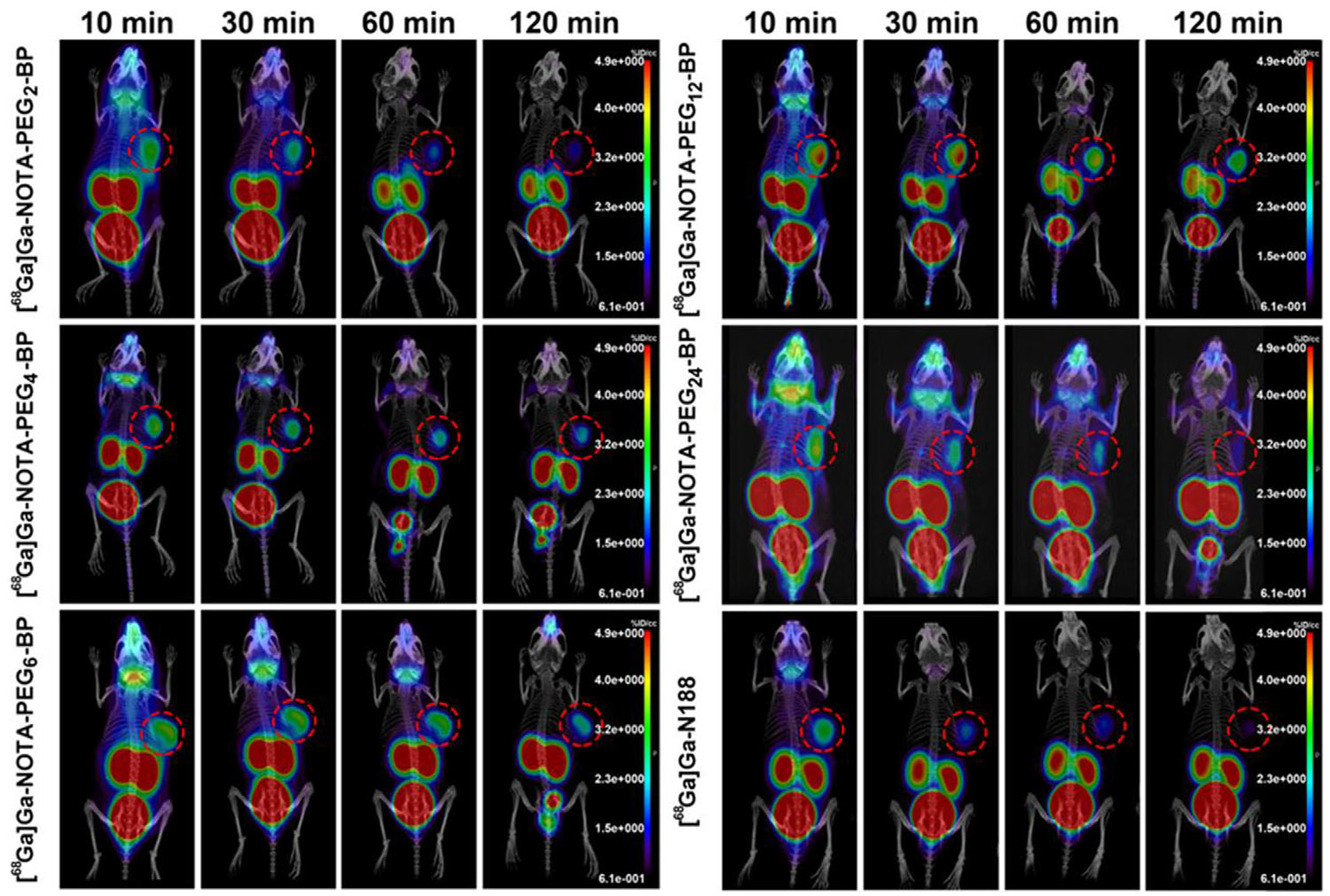
PET/CT images of ^68^Ga–NOTA–PEG_2/4/6/12/24_–BP and ^68^Ga–N188 in SW780 tumor-bearing mice. Reproduced with permission.^106^ Copyright 2024, American Chemical Society. PET, positron-emission tomography.

**FIGURE 7:**
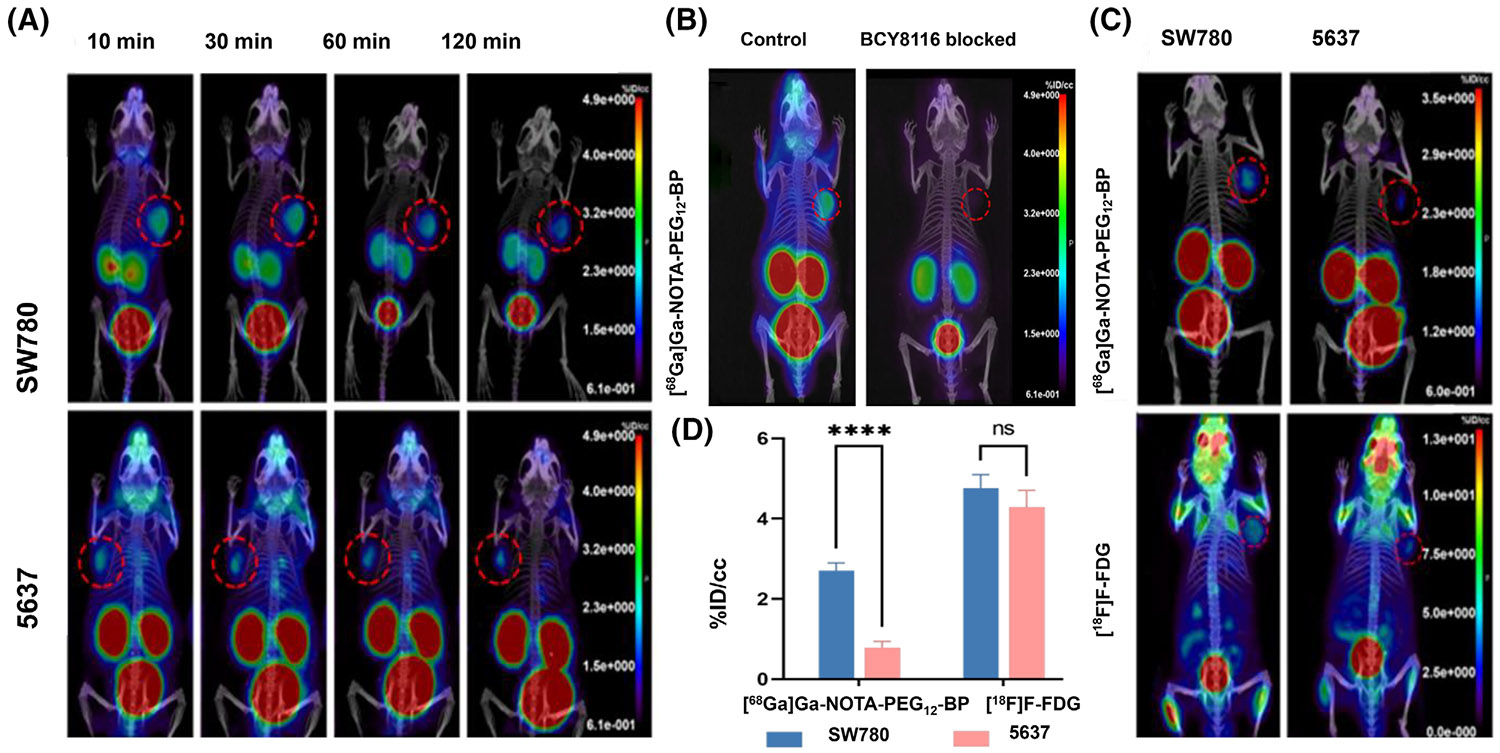
PET/CT images of ^68^Ga–NOTA–PEG_12_–BP and ^18^F-FDG in UC. (A) PET/CT images of ^68^Ga–NOTA–PEG_12_–BP in SW780 and 5637 tumor-bearing mice. (B) ^68^Ga–NOTA–PEG_12_–BP PET/CT images of blocking experiments in SW780 tumor-bearing mice with/without BCY8116 as blocking agents. (C) PET/CT images of ^68^ Ga–NOTA–PEG_12_–BP and ^18^F-FDG in SW780 and 5637 tumor-bearing mice. (D) The tumor uptake of ^68^Ga–NOTA–PEG_12_–BP and ^18^F-FDG in tumor models. Reproduced with permission.^106^ Copyright 2024, American Chemical Society. PET, positron-emission tomography.

After the development of ^68^Ga–N188, the first bicyclic peptide probe for Nectin4-targeted imaging, Wan et al.^106^ also developed a bicyclic peptide probe, ^68^Ga–NOTA– PEG_12_–BP showing good visualization of Nectin4 expression in tumors. They enhanced the metabolism of radioactive tracers in vivo by introducing different amounts of PEG side chains between the bicyclic peptide structure and the chelating agent. They analyzed PET images of five pegylated tracers and found that tumor uptake first increased and then decreased as PEG chains lengthened, reaching a peak at 12 PEG units (^68^Ga–NOTA–PEG_12_–BP) (Figure 6). In subsequent in vivo investigations, PET imaging and blocking studies conducted on SW780 and 5637 xenograft mouse models demonstrated that ^68^Ga–NOTA– PEG_12_–BP displayed superior pharmacokinetic characteristics. These were marked by swift and advantageous tumor accumulation, prompt clearance from background tissues, and enhanced contrast between the tumor and surrounding tissue. The radioactivity accumulation of ^68^Ga–NOTA–PEG_12_–BP in the 5637 tumors was significantly lower than that in the SW780 tumors (Figure 7A), and when BCY8116 was utilized as a competitive blocker, the uptake in the SW780 tumors was significantly blocked at 30 min postinjection (Figure 7B). They then compared the PET/CT imaging of ^18^F-FDG and ^68^Ga–NOTA–PEG_12_– BP tracers in two mice-bearing tumor models (SW780 and 5637) with different Nectin4 expressions (Figure 7C). Although the tumor uptake value of ^68^Ga–NOTA–PEG_12_– BP was slightly lower than ^18^F-FDG, its uptake value was significantly different from that of the two models with different expressions of Nectin4 (SW780: 2.61 ± 0.35% ID/g; 5637: 1.22 ± 0.25% ID/g, *n* = 3,*p* < .0001), and there was no significant difference in ^18^F-FDG uptake value (Figure 7D). The results showed that PET images of bicyclic peptide probes were able to distinguish between high and low Nectin4 expression levels in tumor tissue, which was consistent with subsequent biological distribution and histological staining results. In addition, the high tumor– background ratio is an ideal feature for cancer imaging tracers, which can provide higher sensitivity for lesion detection.^107^ In this study, ^68^Ga–NOTA–PEG_12_–BP uptake in SW780 tumors was 2–3 times higher than ^68^Ga–N188, effectively reducing background radioactivity. This results in much greater contrast between the tumors in the blood, liver, and muscle 60 min after injection (^68^Ga–NOTA– PEG12–BP vs. ^68^Ga-N188: T/B 8.33 ± 1.90 vs. 0.58 ± 0.10; T/L 11.03 ± 1.28 vs. 1.15 ± 0.31; T/M 22.89 ± 8.94 vs. 6.23 ± 1.99). Therefore, ^68^Ga–NOTA–PEG_12_–BP has strong development potential as a new radiotracer for PET imaging of bladder cancer.

**FIGURE 8:**
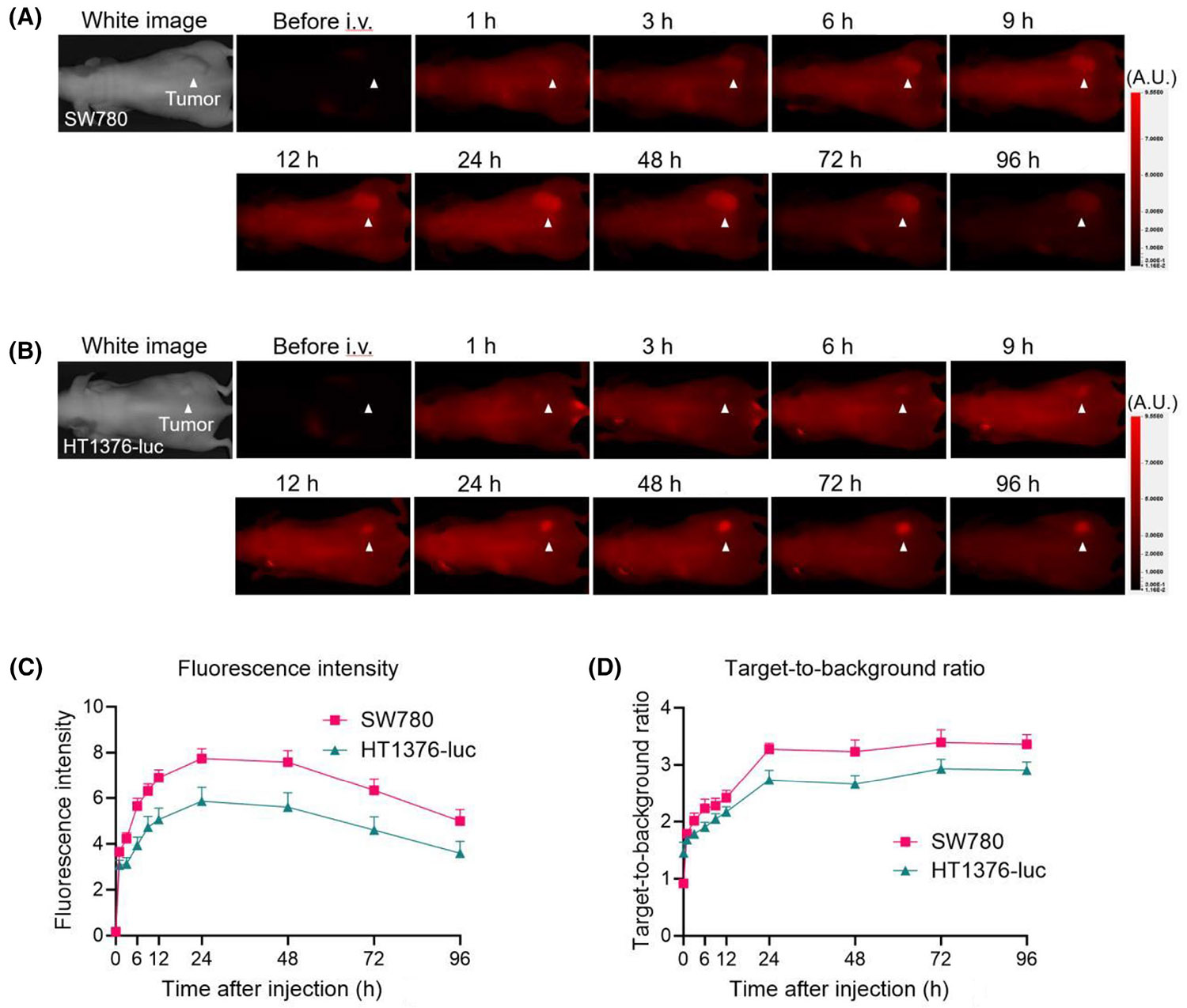
NIRF images and imaging analysis of Nectin4–IR700. The NIRF images in a SW780 (A) and HT1376-luc (B) tumor-bearing mouse. Quantitative analysis of fluorescence intensity (C) and target-to-background ratio (D) at the tumor site after infusing Nectin4–IR700 (*n* = 10). Reproduced with permission.^108^ Copyright 2024, Elsevier. NIRF, near-infrared fluorescence.

In addition to radionuclide-labeled probes, fluorescent probes can also be used to detect bladder cancer. Fukushima et al.^108^ combined a Nectin4-targeted mAb with the NIR silicon phthalocyanine dye, IRDye700DX (IR700) to form a fluorescent probe Nectin4–IR700. They performed continuous in vivo NIRF imaging in SW780 and HT1376-luc tumor models after injection of Nectin4–IR700 (Figure 8A,B). It was found that the intra-tumor FL intensity of Nectin4–IR700 in both models reached a peak at 24 h and then gradually decreased. The target–background ratio (TBR) of Nectin4–IR700 exhibited an increase within 24 h postinjection, subsequently stabilizing across both models throughout the observation period. Notably, the FL intensity and TBR of Nectin4–IR700 in SW780 tumors appeared to be elevated compared with those in HT1376-luc tumors (Figure 8C,D), which was consistent with the expression level of Nectin4 in vivo. These results suggest that IR700 FL is visible in situ in bladder tumors and NIRF imaging based on Nectin4–IR700 may help optimize tumor detection during surgery and carefully evaluate excision margins.

In general, mAb, ADC, and bicyclic peptides have been used as tracers for diagnostic imaging of UC. Both ^68^Ga–N188, ^124/125^I-EV, ^68^Ga–NOTA–PEG_12_–BP for PET/CT imaging and Nectin4–IR700 for NIRF imaging have demonstrated the potential to identify Nectin4-positive tumors and are expected to be powerful tools for tumor diagnosis. Because of the large molecular weight of mAbs and the slow blood clearance, bicyclic peptide probes with smaller molecular weight have more potential for development. Notably, these probes are excreted through the urinary system, resulting in high background radioactivity that is not conducive to the UC diagnosis. Nevertheless, this radiotracer remains important for the accurate assessment of metastatic bladder cancer lesions or other tumors with high Nectin4 expression, such as TNBC and pancreatic cancer. Considering the necessity of detecting UC lesions, future research focuses on improving the affinity of probes to Nectin4 and reducing the background radioactivity.

### 4.3 ∣ Gastric cancer

**FIGURE 9:**
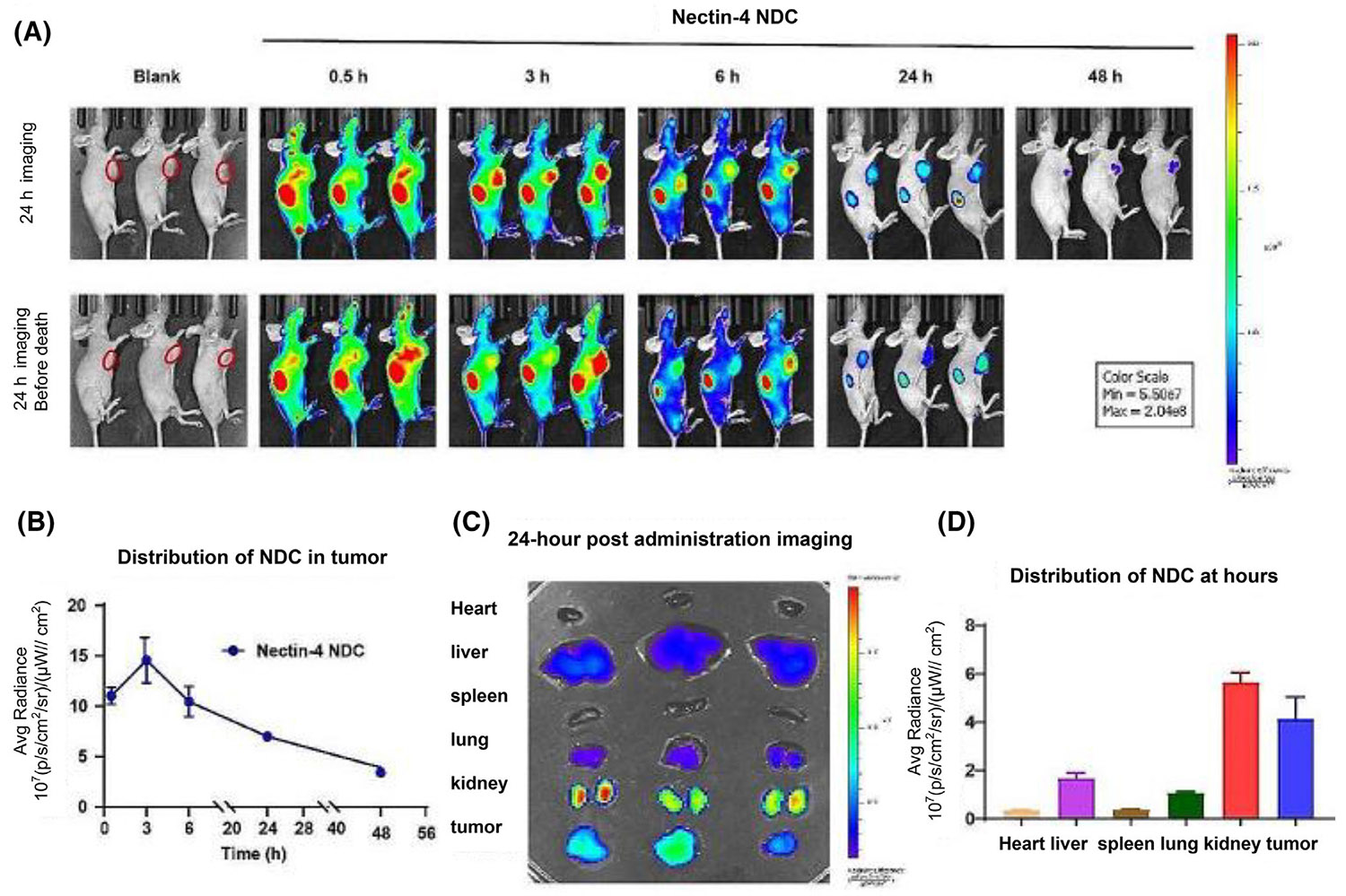
In vivo distribution and tumor uptake of Nectin4 NDC in gastric cancer. (A) Fluorescence imaging of mice injected with Cy7–Nectin4 NDC at different times. (B) The average fluorescence radiance values of NDC in tumor. (C) Fluorescence distribution in major organs at 24 h postinjection. (D) Quantitative analysis of the average fluorescence intensity (*n* = 3). Reproduced under the terms of the CC-BY 4.0 license.^111^ Copyright 2024, Springer. NDC, nanobody drug conjugates.

Gastric cancer, a primary epithelial malignant tumor originating in the stomach, is one of the most common cancers and the third leading cause of cancer-related death globally.^109^ The high expression of Nectin4 is closely related to the differentiation, lymph node metastasis, and TNM stage of gastric cancer.^110^ This makes Nectin4 a potential target for the diagnosis and prognostic prediction of gastric cancer. Wu et al.^111^ used Cyanine7 (Cy7)-labeled Nectin4–NDC as a fluorescent probe to perform NIRF imaging in BALB/c nude mice bearing NCI–N87 gastric cancer xenografts. Cy7 is a NIRF dye with a high extinction coefficient, high stability, and high FL intensity.^112^ They observed that Cy7–Nectin4 NDC exhibited rapid systemic tissue distribution in vivo, as well as significant uptake in tumors and kidneys (Figure 9A). By observing the images and analyzing the average radiation efficiency of the tumor area, it was found that Nectin4 NDC reached the peak accumulation at the tumor site at 3 h, then gradually cleared (Figure 9B), and stayed in the tumor site for a longer time than other sites. Subsequent FL imaging results of mouse tissue in vitro showed that FL mainly accumulated in the tumor and kidney, and relatively less in the liver and lung, which was consistent with the results of in vivo imaging (Figure 9C,D). These demonstrated that Nectin4 NDC has excellent in vivo targeting ability, stays at tumor sites longer than other sites, and has the potential of NIRF imaging to guide surgery.

### 4.4 ∣ Pancreatic cancer

Pancreatic cancer is one of the most lethal malignancies, and despite accounting for 5% of all cancer cases, it is the second leading cause of cancer-related death worldwide.^113^ The important reason for the poor prognosis is that the disease symptoms are not obvious, leading to late diagnosis and early metastasis.^114^ It has been found that Nectin4 is overexpressed in pancreatic cancer, which is associated with larger tumor volume, tumor proliferation, and angiogenesis.^20,115^ In addition, the expression level of Nectin4 is low in normal adult tissues, which makes Nectin4 a potential biomarker for the early diagnosis of pancreatic cancer.

After the first Nectin4-targeted bicyclic peptide probe ^68^Ga–N188 was used for diagnostic imaging of UC, the team went on to use the probe in 62 patients with 16 cancer types.^104^ They performed ^68^Ga–N188 PET/CT on these 62 patients, the largest sample size of which was pancreatic cancer (*n* = 19), and found that its average SUVmax was the second highest among the 16 tumors. They then collected tumor samples from 36 patients (including 14 patients with pancreatic cancer) to investigate the correlation between SUVmax and Nectin4 expression. A significant positive correlation was found between SUVmax and membrane Nectin4 expression (*r* = 0.458, *p* = .005), but there was no significant correlation between SUVmax and cytoplasmic Nectin4 expression (*r* = 0.033, *p* = .847). These results demonstrated that the SUVmax was high in pancreatic cancer, and there was a significant positive correlation between SUVmax and membrane Nectin4 expression. In a follow-up comparison of ^18^F-FDG and ^68^Ga–N188, the detection rate of ^68^Ga–N188 (100.00%) was higher than ^18^F-FDG (66.67%) in the assessment of residual/recurrent pancreatic cancer. ^68^Ga–N188 detected four patients with residual or recurrent pancreatic tumors missed by ^18^F-FDG, and two additional patients with pancreatic cancer missed by ^18^F-FDG with additional lymph node and liver metastases. All these indicate that ^68^Ga–N188 has a higher diagnostic detection rate than ^18^F-FDG in pancreatic cancer, and it has advantages in the restaging of lesions after neoadjuvant chemotherapy and surgery. In conclusion, ^68^Ga–N188 imaging has the potential to improve the detection rate of pancreatic cancer, predict clinical outcomes, and help clinical decision-making.

### 4.5 ∣ Other solid tumors

In a recent study using ^68^Ga–N188 PET/CT in multiple different cancers, a total of 16 tumors were included.^104^ In addition to UC (*n* = 16) and pancreatic cancer (*n* = 19), which had the largest sample sizes, there were also 14 other cancers including ovarian cancer, cervical cancer, colon cancer, non-small cell lung cancer, and so on. The study found that ^68^Ga–N188 had a higher average SUVmax in these 62 patients with different cancer types. It was also found that SUVmax was only positively correlated with membrane Nectin4 expression, but not with cytoplasmic Nectin4 expression in primary and metastasis samples from 36 patients. These confirmed the feasibility of non-invasive and quantitative PET imaging to obtain systemic tumor load and Nectin4 status and may guide personalized treatment strategies.

Then they compared ^18^F-FDG with ^68^Ga–N188 based on the number of patients, tumor lesions, and lymph nodes detected. For patient-based comparisons, the detection rates of ^68^Ga–N188 (95.00% [57/60]) and ^18^F-FDG PET/CT (93.33% [56/60]) were comparable, with no statistical difference. For lesion-based comparison, ^18^F-FDG detected all 45 primary tumors (100.00% [45/45]) and ^68^Ga–N188 detected 39 primary tumors (86.67% [39/45]) (*p* = .031). The six tumors missed by ^68^Ga–N188 were classified into four types: UC, intrahepatic cholangiocarcinoma, cervical cancer, and hepatocellular carcinoma. However, ^68^Ga–N188 was able to detect all 12 residual or locally recurrent tumors (100.00% [12/12]), but ^18^F-FDG failed to identify 4 pancreatic cancers (66.67% [8/12]) (*p* = .125). In lymph node detection, the detection rate of ^18^F-FDG PET/CT was higher than that of ^68^Ga–N188 PET/CT (94.62% [88/93] vs. 76.34% [71/93] [*p* < .001] for lymph node metastases and 96.83% [61/63] vs. 71.43% [45/63] [*p* < .001] for distant metastases). In general, the sensitivity and accuracy of ^18^F-FDG PET/CT (*p* < .001) were higher than ^68^Ga–N188 PET/CT (*p* = .002). The specificity of ^68^Ga–N188 PET/CT was higher than that of ^18^F-FDG PET/CT (63.64% [7/11] vs. 36.36% [4/11]), although the difference did not reach statistical significance (*p* = .250). In addition, TNM staging or restaging based on ^68^Ga–N188 and ^18^F-FDG PET/CT were consistent in 58 of 62 patients. In two patients with pancreatic cancer with additional lymph nodes and liver metastases missing from ^18^F-FDG, ^68^Ga–N188 upstaged the TNM stage. In contrast, ^18^F-FDG PET/CT upstaged one patient with intrahepatic cholangiocarcinoma and one patient with ovarian cancer because ^68^Ga–N188 missed lymph node metastasis. This suggests that ^68^Ga–N188 and ^18^F-FDG PET/CT can complement each other to reduce missed diagnoses.

In conclusion, in this study of 62 patients with 16 different types of cancer, a significant positive correlation was found between SUVmax and membrane Nectin4 expression. ^68^Ga–N188 can detect some tumor lesions missed by ^18^F-FDG, but the potential advantages over ^18^F-FDG need further studies. There are still certain limitations, such as a small sample size, an unrepresentative SUVmax, and a short patient follow-up time. Future clinical studies may increase the sample size and follow-up time to investigate patient outcomes and fully explore the role of ^68^Ga–N188 as an imaging biomarker.

## 5 ∣ CONCLUSION

Nectin4 is frequently overexpressed in a range of solid tumors and strongly correlates with cell proliferation, metastasis, angiogenesis, adhesion, recurrence, DNA mismatch repair, drug resistance, and adverse prognosis.^20^ The US FDA has approved EV, an ADC targeting Nectin4, for treating UC, indicating the importance of Nectin4-targeted therapy. In addition, accurate diagnosis and evaluation of patients are critical. Therefore, the development of Nectin4-targeted tracers to identify patients with high Nectin4 expression is significant for tumor diagnosis and selecting patients suitable for EV therapy.

Nectin4-targeted molecular imaging can be divided into optical and nuclear medicine imaging, providing diverse options for diagnostic applications. The main application of optical imaging in Nectin4 targeting is NIRF. This technique uses NIRF dyes such as ICG, Cy7, and IR700 to label anti-Nectin4 mAbs or NDCs as fluorescent probes to perform FL imaging to reveal Nectin4-positive tumors. It can be used for cancer imaging and surgical guidance to ensure complete removal of tumor tissue.^116^ However, its limited tissue penetration restricts utility to superficial or surgically exposed lesions. In contrast, nuclear medicine imaging offers systemic, deep-tissue visualization, critical for detecting metastases and assessing Nectin4 expression across tumor sites. Some studies use radionuclides with long half-lives, such as ^89^Zr and ^124/125^I, to label Nectin4-targeted mAbs and ADCs, because these probes have large molecular weights, low blood clearance, and long circulation time, requiring longer detection times for clinical applications.^71^ Therefore, the development of smaller molecular probes is the trend of research. Bicyclic peptides labeled with short half-life nuclide ^68^Ga have been developed, which can better meet the needs of clinical practice as small molecule probes.^63^ Currently, the bicyclic peptide probe ^68^Ga–N188 has been clinically studied in UC and various other tumor patients, marking significant progress in the Nectin4-targeted diagnostic probes. However, the probe has low tumor uptake and high background radioactivity, indicating its sensitivity is not ideal. Fortunately, the ^68^Ga–NOTA–PEG_12_–BP and ^68^Ga–DN68 bicyclic peptide probes developed significantly improved the tumor–background ratio, providing higher sensitivity for focal detection.^117^

However, these probes are mainly metabolized by the urinary system, and high background radioactivity can interfere with the diagnosis of UC. Future research may focus on further modifying its structure and changing its metabolic pathway (e.g., hepatic clearance) to improve the diagnostic effect of UC. In addition, improving the affinity of the molecular probes to Nectin4 is also a major research direction. To facilitate the development of Nectin4-targeted diagnosis and therapy, the construction of humanized Nectin4 mouse models and the expansion of clinical trials are critical. These efforts will rigorously evaluate the imaging efficacy of Nectin4 tracers in solid tumors, paving the way for more personalized and precise cancer treatments.

## Figures and Tables

**TABLE 1:** Theranostic applications of different molecular imaging targeting Nectin4.

Types of cancer	Molecular imaging	Imaging agents	Tumor model	Findings	References
Breast cancer	ImmunoPET	^89^Zr/^18^F-AGS- 22M6	MDA–MB-231 xenograft breast cancer and AG-B8/AG-B7/AG- Mel5/SW780 PDX	^89^Zr–AGS-22M6 can be used to distinguish tumors based on Nectin4 expression. Tumors in the liver and bone were detected and differentiated based on Nectin4 expression.	Campbell et al.^89^
	ImmunoSPECT/CT NIRF	^99m^Tc–HYNIC– mAb_Nectin4_ and mAb_Nectin4_–ICG	MDA–MB-468 xenograft TNBC	^99m^Tc–HYNIC–mAb_Nectin4_ SPECT/CT imaging is useful for delineating Nectin4 expression in TNBCs and promotes precision diagnosis and classification. mAb_Nectin4_–ICG has excellent tumor-specific targeting and long-term retention.	Shao et al.^92^
	PET/CT	^68^Ga–DN68	MDA–MB-468 xenograft TNBC	^68^Ga–DN68 can be used as a radioactive tracer for visualizing Nectin4-positive TNBC. In addition, the PET imaging capability of ^68^Ga–DN68 is expected to quantify Nectin4.	Ge et al.^97^
Urothelial carcinoma	PET/CT	^68^Ga–N188	SW780/5637 xenograft urothelial carcinoma and patients with advanced UC	A clear correlation between PET SUV value and Nectin4 expression was observed, supporting the application of ^68^Ga–N188 PET as a companion diagnostic tool for optimizing treatments that target Nectin4.	Duan et al.^102^
	PET/CT	^124/125^I-EV	SW780/T24 xenograft urothelial carcinoma	^124^I-EV was successfully prepared with high specificity and binding affinity of Nectin4. This radioactive probe completely simulates the internal circulation of ADC drugs and tumor uptake and retention.	Ren et al.^60^
	PET/CT	^68^Ga–NOTA– PEG12–BP	SW780/5637 xenograft urothelial cancer	^68^Ga–NOTA–PEG12–BP shows excellent visualization of Nectin4 expression in tumors, with the advantages of high tumor uptake, long tumor retention time, and minimal background activity.	Wan et al.^106^
	NIRF	Nectin4–IR700	SW780/HT1376-luc xenograft bladder cancer	Orthotopic bladder tumors clearly showed IR700 fluorescence. Nectin4–IR700 may help detect bladder tumors during operation.	Fukushima et al.^108^
Gastric cancer	NIRF	Cy7–Nectin4 NDC	BALB/c nude mice bearing NCI-N87 human gastric cancer xenografts	Cy7-labeled Nectin4 NDC exhibits high levels of tumor uptake and tumor targeting, and its binding with NIRF has the potential to serve as a specialized tracer for cancer imaging.	Wu et al.^111^
Various cancers	PET/CT	^68^Ga–N188 and ^18^F-FDG	62 patients with 16 different types of cancer	A significant positive correlation was found between SUVmax and membrane Nectin4 expression (*r* = 0.458, *p*= .005). ^68^Ga–N188 can detect some tumor lesions missed by ^18^F-FDG.	Zhang et al.^104^

Abbreviations: PET, positron-emission tomography; SPECT, single-photon emission computed tomography; ICG, indocyanine green; NIRF, near-infrared fluorescence; ADC, antibody–drug conjugate; PDX, patient-derived xenograft; SUV, standard uptake value.
